# Aquaporins 8 and 9 as Possible Markers for Adult Murine Lacrimal Gland Cells

**DOI:** 10.1155/2021/6888494

**Published:** 2021-09-09

**Authors:** Naoko Okada, Tetsuya Kawakita, Masataka Ito, Kazuo Tsubota

**Affiliations:** ^1^Department of Ophthalmology, Keio University School of Medicine, Tokyo, Japan; ^2^Department of Pharmaceutical Sciences, Nihon Pharmaceutical Hospital, Saitama, Japan; ^3^Department of Ophthalmology, Kitasato University Kitasato Institute Hospital, Tokyo, Japan; ^4^Department of Developmental Anatomy and Regenerative Biology, National Defense Medical College, Tokorozawa, Japan

## Abstract

Aquaporins (AQPs) are proteins that selectively transport water across the cell membrane. Although AQPs play important roles in secretion in the lacrimal gland, the expression and localization of AQPs have not been clarified yet. In the current study, we investigated the expression pattern of AQP family members in the murine lacrimal gland during development. Lacrimal gland tissues were harvested from E13.5 and E17.5 murine embryos and from mice 8 weeks of age (adults). Corneal and conjunctival tissues from the latter served as controls. Total RNA was isolated and analyzed for the expression of AQP family members using qPCR. The localization of AQPs in the adult lacrimal gland in adult murine lacrimal glands was also analyzed. Expression of *Aqp*8 and *Aqp*9 mRNAs was detected in the adult lacrimal gland but not in the cornea, conjunctiva, or fetal lacrimal gland. AQP8 and AQP9 and *α*-SMA partially colocalized around the basal regions of the acinar unit. The levels of *Aqp*3 mRNAs and protein were much lower in the adult lacrimal gland but were readily detected in the adult cornea and conjunctiva. Our study suggests that AQP8 and AQP9 may serve as markers for adult murine lacrimal gland, ductal, and myoepithelial cells.

## 1. Introduction

The integrity of the ocular surface depends greatly on the qstability of the film of tears that covers the anterior surface of the eye. The formation of this film depends in part on the secretory function of the lacrimal gland, which continuously secretes tear fluid. Tears contain proteins, enzymes, lipids, and metabolites that moisten, lubricate, and protect the surface of the eye [1]. Several lacrimal gland dysfunctions such as Sjögren's syndrome induce sight-threatening keratinization of the ocular surface epithelium, which causes corneal infection, irritation, and scarring [2, 3]. These studies indicate that the secretory function of lacrimal gland is important for understanding the chronic disabling condition called dry eye syndrome.

The mechanism of fluid secretion by the lacrimal gland is similar to that of the salivary and other exocrine glands. Active pumping of ions into the gland's acinus is followed by passive, osmotically driven water flow to generate a nearly isotonic ultrafiltrate of plasma. The fluid expelled from the acinar lumen is further modified by the transport of electrolytes, water, and proteins by ductal cells to produce an approximately isotonic tear fluid enriched in potassium and chloride. Aquaporin (AQP) channels transport water against osmotic gradients across the plasma membranes of many cells, including those in the eye [4].

Aquaporins (AQPs) are transmembrane proteins that selectively and rapidly transport water across the cell membrane [5]. There are at least 13 AQP family members (AQP0–AQP12) in mammalian cells [6]. AQPs are expressed in the epithelial and endothelial layers of various organs involved in fluid transport such as kidney tubules and glandular epithelia [7, 8] as well as in cell types that do not transport fluid such as the skin [9] and fat cells [10]. In most cell types, the AQPs reside constitutively in the plasma membrane.

The AQPs of ocular and extraocular tissues are critical to physiological function and play roles in pathophysiology [11]. In the lacrimal gland, AQP5, AQP4, and AQP1 are expressed in the apical membranes of acinar and duct cells, in the contralateral basolateral membranes [12], and in surrounding microvascular endothelia [13], respectively. AQP1 has been reported to be related with human and mouse corneal endothelial dysfunction [14]. AQP5-null mice produce low volumes of hypertonic and highly viscous saliva [15, 16]. Whereas tear secretion in *Aqp*5-null mice is normal [13, 17], corneal epithelial wound healing was delayed compared with that in wild-type [18]. Moreover, our own studies show that the localization of AQP5 in patients with Sjögren's syndrome influences the secretory function of the lacrimal gland [19]. Therefore, we hypothesized that the expression and localization of AQPs play important roles in normal secretion by the lacrimal gland and in dry eye syndrome.

AQPs are distributed in a tissue-specific pattern and are temporally expressed in a certain organ [20, 21]. Despite their morphological differences, the cornea, conjunctiva, and lacrimal gland of the vertebrate eye originate from the surface ectoderm of the developing head during embryonic development. Murine lacrimal gland development begins on embryonic day (E)13.5 as a tubular invagination of the conjunctival epithelium at the temporal extremity of the eye [22]. After a period of elongation, the lacrimal gland bud invades the mesenchymal sac on E16.5 and begins a period of rapid growth and branching to form prospective lobular structures called the intra- and exorbital lobes. During the postnatal period, lacrimal glands continue to branch and differentiate, and by the time of eye reopening seven days after birth, they have begun to produce a secretion containing mucins, lipids, lysozyme, and immunoglobulins [23], which lubricates and protects ocular surfaces.

The contribution of AQP family members to the development of lacrimal gland is unknown. Therefore, this study is aimed at determining the patterns of expression of *Aqp*09, *Aqp*11, and *Aqp*12 during the pre- and postnatal development of the murine lacrimal gland. The levels of *Aqp* mRNAs that were expressed at detectable levels were compared with those expressed in adult murine corneal and conjunctival tissues. The intracellular localization of selected AQPs in the lacrimal glands was determined using immunohistochemistry.

## 2. Results

### 2.1. Expression of *Aqp* mRNAs in the Adult Murine Conjunctiva, Cornea, and Lacrimal Gland

**Figure 1:**
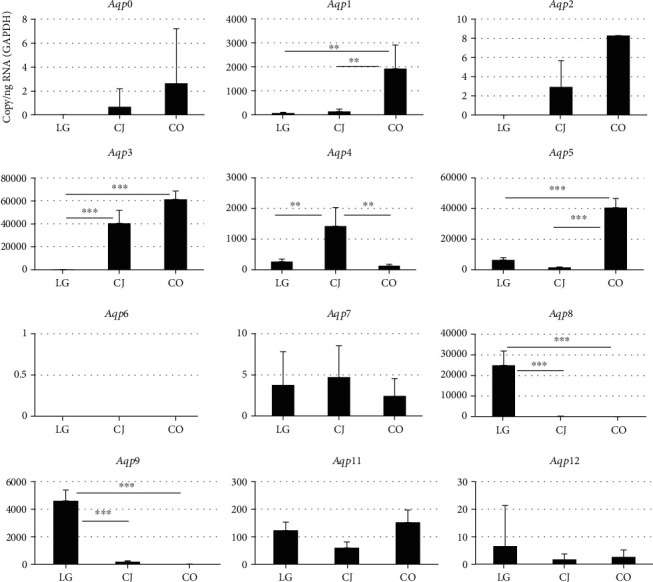
Analysis of expression of *Aqp* family members of mRNAs in an adult mouse lacrimal gland, conjunctiva, and cornea. Expression of each *Aqp* mRNA in an adult lacrimal gland (LG), conjunctival (CJ), and corneal (CO) tissues was shown. Total RNAs isolated from these three tissues were subjected to qPCR analysis to detect the expression of *Aqp*0-*Aqp*9, *Aqp*11, and *Aqp*12. Data were normalized to that of *Gapdh* (^∗^*p* ≤ 0.05, ^∗∗^*p* ≤ 0.01, and ^∗∗∗^*p* ≤ 0.001).

We compared the levels of *Aqp* mRNAs in the adult murine conjunctiva, cornea, and lacrimal gland. The expression levels of *Aqp*0, *Aqp*2, *Aqp*6, *Aqp*7, *Aqp*11, and *Aqp*12 were very low in all of adult murine conjunctiva, cornea, and lacrimal gland (Figure 1), and there were no significant differences among adult murine conjunctiva, cornea, and lacrimal gland. *Aqp*1 mRNA levels were significantly higher in the corneal than in the conjunctiva and lacrimal gland (Figure 1, *p* ≤ 0.01 and *p* ≤ 0.01, respectively). *Aqp*3 mRNA was detected at significantly higher levels in the cornea and conjunctiva compared with the lacrimal gland (Figure 1, *p* ≤ 0.001 and *p* ≤ 0.001, respectively). *Aqp*4 mRNA was detected at significantly higher levels in the conjunctiva compared with in the cornea and lacrimal gland (Figure 1, *p* ≤ 0.01 and *p* ≤ 0.01, respectively). The expression of *Aqp*5 mRNA was significantly higher in the cornea than in the conjunctival and lacrimal gland (Figure 1, *p* ≤ 0.001 and *p* ≤ 0.001, respectively). Significantly higher levels of *Aqp*8 and *Aqp*9 mRNAs were detected in the adult lacrimal gland tissue than the in cornea and conjunctiva (Figure 1, *p* ≤ 0.001 and *p* ≤ 0.001, respectively).

### 2.2. Analysis of *Aqp* mRNA Expression in Fetal and Adult Murine Lacrimal Glands

**Figure 2:**
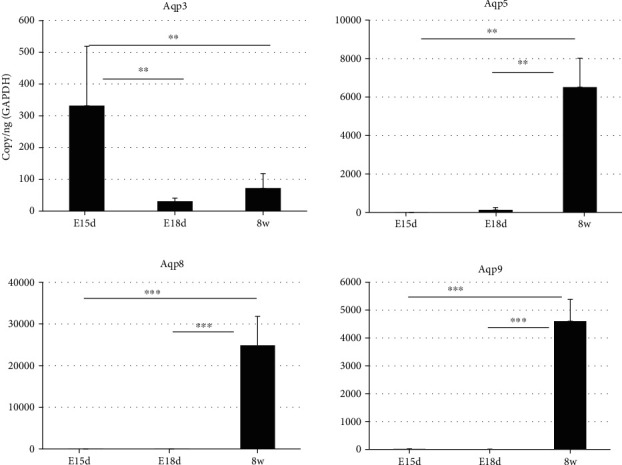
Analysis of *Aqp* family members of mRNA expression during the development of murine lacrimal gland. Expression of individual *Aqp* mRNAs (*Aqp*3, *Aqp*5, *Aqp*8, and *Aqp*9) measured on E13.5 and E17.5 and in adult lacrimal gland tissues using qPCR was shown (^∗^*p* ≤ 0.05, ^∗∗^*p* ≤ 0.01, and ^∗∗∗^*p* ≤ 0.001).

We next selected *Aqp*3, *Aqp*5, *Aqp*8, and *Aqp*9 mRNA for further analysis to investigate the potential specific makers for adult lacrimal gland based on the expression level and pattern in Figure 1. The expression levels of *Aqp*3, *Aqp*5, *Aqp*8, and *Aqp*9 mRNA in lacrimal glands at E13.5 (budding morphogenesis stage of the lacrimal gland) and E17.5 (branching morphogenesis stage) and in adults were compared. *Aqp*3 mRNA expression was relatively low but significantly higher on E13.5 than on E17.5 and 8 w (Figure 2, *p* ≤ 0.01 and *p* ≤ 0.01, respectively). *Aqp*5 mRNA expression was relatively high and significantly higher on 8 w than on E13.5 and E17.5. (Figure 2, *p* ≤ 0.01 and *p* ≤ 0.01, respectively). *Aqp*8 and *Aqp*9 mRNA expressions were relatively high and significantly higher on 8 w than on E13.5 or E17.5 (Figure 2, *p* ≤ 0.001 and *p* ≤ 0.001, respectively).

*Aqp* mRNA expression in the murine lacrimal glands (fetal and adult), adult cornea, and conjunctiva were summarized in Table 1. *Aqp5*, *Aqp8*, and *Aqp9* mRNA expressions were relatively high in the adult lacrimal gland.

### 2.3. IFA of AQP Expression in Adult Murine Lacrimal Gland Tissues

**Figure 3:**
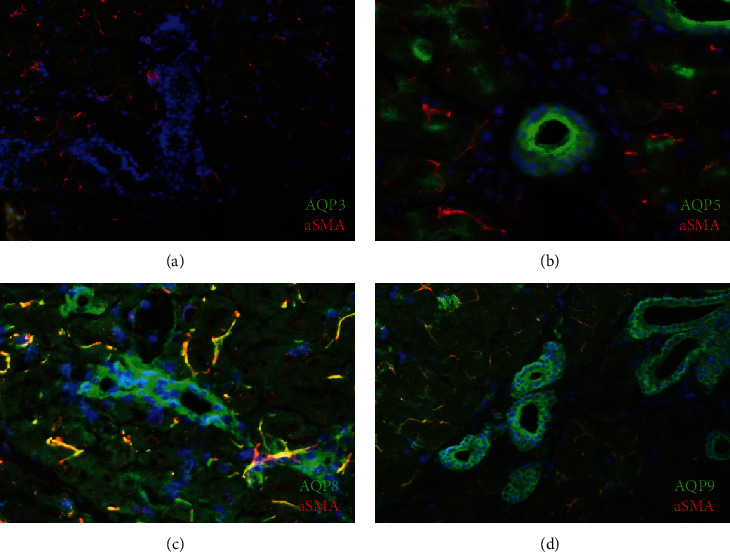
IFA of AQP3, AQP5, AQP8, and AQP9 expressions in adult murine tissues. IFA of AQP3 (a, green), AQP5 (b, green), AQP8 (c, green), and AQP9 (d, green) and *α*-SMA (a–d, red) in adult lacrimal gland tissues was shown (magnification, 200x). Nuclei were stained with DAPI (blue).

The localization of AQP3, AQP5, AQP8, and AQP9 was analyzed in lacrimal glands using IFA. AQP3 expression was not detected in either acinar or ductal cells in the lacrimal gland (Figure 3(a)). AQP5 expression was detected in the apical region of the cytoplasm of both ductal and acinar cells in the lacrimal gland (Figure 3(b)). In contrast, intense expression of AQP8 was detected in the basal region of lacrimal gland ductal cells rather than acinar cells (Figure 3(c)). AQP9 expression was also observed in the basal region of lacrimal duct cells (Figure 3(d)). Moreover, AQP8 and AQP9 expression was detected in myoepithelial cells whose processes form arcs around the acinar cells of the lacrimal gland. AQP8 or AQP9 (green) and *α*-SMA (red) were partially colocalized (yellow) around the basal regions of the acinar unit, consistent with the localization of AQP8 or AQP9 in myoepithelial cells. AQP5 (green) did not colocalize with *α*-SMA (Figure 3(b)). These results suggested that AQP8 and AQP9 were localized to the basal region of ductal and myoepithelial cells in the lacrimal gland.

## 3. Discussion

The transport of water mediated through the lacrimal glands is essential for maintaining corneal stability and homeostasis of the ocular surface, and AQPs are essential for this process to control the water permeability of membranes. The present results demonstrate that *Aqp*8 and *Aqp*9 expression was specific to the adult murine lacrimal gland and was not detected in the cornea and conjunctiva by qPCR. IFA demonstrated that their expression levels were higher in the epithelial basal layers of the duct than in the acinus. Interestingly, AQP8 or AQP9 and *α*-SMA were coexpressed in myoepithelial cells around the lacrimal gland acinar units. In contrast, AQP5 expression was observed higher in the apical layers of ductal epithelium and acinar cells. AQP3 expression was observed in the lacrimal gland on E13.5, but then decreased with differentiation, while high expression was confirmed in the adult cornea and conjunctival tissue. Taken together, our results suggest that AQP family members were expressed in a tissue-specific manner during development.

In the present study, we detected *Aqp*8 mRNA and protein in the adult lacrimal gland. AQP8 is expressed in the liver, pancreas, salivary glands, testis, colon, kidney, and airway in mammal [24–26], suggesting that AQP8 is required for the secretion of saliva, bile, and pancreatic fluid and intestinal fluid absorption/secretion and for concentrating urine. From biopsy samples of patients, AQP8 expression was permanently downregulated in ulcerative colitis patients compared to noninflammatory bowel disease subjects, which may be involved in pathological abnormalities in absorptive function in the intestinal tract [27]. However, contrary to expectations from the AQP8 expression pattern and from results of studies of transgenic mice lacking other *Aqp*s, the phenotype of *Aqp*8-null mice exhibited only minor phenotypic differences between wild-type and *Aqp*8-null mice, even after attempting to expose subtle phenotypic differences by physiological stress and deletion of other aquaporins [28]. Although there is no direct evidence to lacrimal gland secretion in this paper, AQP8 may not directly be involved in transcellular water transport in the murine lacrimal gland. Recently, Calamita et al. showed that AQP8 was expressed in the inner mitochondrial membrane of hepatocytes and mediates water and ammonia transport, which may be particularly important for the rapid expansion of mitochondrial volume and detoxification, such as those that occur during active oxidative phosphorylation and following apoptotic signals [29, 30]. Thus, it cannot be excluded that AQP8 mediates functions other than water transport in the murine lacrimal gland.

We also show here for the first time, to our knowledge, the expression of AQP9 in the lacrimal gland. AQP9 is a member of the aquaglyceroporin subfamily of AQPs and shares the highest amino acid sequence similarity with AQP3, AQP7 [31]. Aquaglyceroporin transports small uncharged molecules such as glycerol, urea, purine, pyrimidine, and arsenite as well as water [31–33] and is expressed in the testes, leukocytes, brain, and liver. In hepatocytes, it resides on plasma membranes facing the sinusoids [34–36], and it may play an important role in hepatic glycerol metabolism and in glycerol and glucose metabolism in patients with diabetes mellitus [37–39]. Moreover, there is evidence that in astrocytes and catecholaminergic neurons, AQP9 contributes to the regulation of energy metabolism in the cranial nerve system [40]. In retina, AQP9 was mainly expressed in the ganglion cell layer and increased intraocular pressure significantly reduced AQP9 expression in the optic nerve head and ganglion cell layers, suggesting the involvement to the pathogenesis of glaucomatous optic neuropathy [41]. Furthermore, AQP9, selectively expressed in catecholaminergic neurons, had been shown to infiltrate parkinsonogenic toxins, which is important in the pathophysiology of Parkinson's disease [42]. Whether AQP9 contributes to the embryonic development and adult function of the lacrimal glands of mice and humans will require further research.

The *Aqp*8 and *Aqp*9 mRNA expression in this study was different from previous studies, which showed *Aqp*9 mRNA and protein expression were not found in the adult rat lacrimal gland [43]. In rat salivary glands, the basolateral localization of AQP8 in acinar cells, coupled with the apical localization of AQP5, has been reported to allow water to cross the acinar epithelium through these channels during primary saliva formation [44]. Our present study showed that AQP8 or AQP9 localized to the basolateral membrane of the duct cells in the murine lacrimal gland, but no expression in acinar cells. Although the species and the age of the animal were different in the previous studies, further studies were needed to confirm.

In this present study, the expression of AQP5 was observed in the adult cornea, conjunctiva, and lacrimal glands and was particularly abundant in the adult cornea (Figure 1). AQP5, AQP8, and AQP9 were detected in adult lacrimal glands but not in fetal lacrimal glands (Figure 2). These findings reveal that AQP5 as well as AQP8 and AQP9 is associated with the functional maturation of lacrimal glands during development. IFA revealed that AQP5 localized to the apical membrane in acinar epithelial and duct cells in lacrimal glands, similar to salivary and airway submucosal glands and lacrimal gland in previous reports [12]. So far, AQP5 had been shown to be closely associated with eye disease. In Sjögren's syndrome, abnormal localization of AQP5 in the lacrimal glands had been reported to contribute to tear loss and dry eye in these patient [19]. In addition, AQP5 expression on conjunctival cells was significantly reduced in post-vitreo-retinal surgery, suggesting that this alteration was associated with dry eye outcome after the surgery [45]. In contrast, the expression of AQP3 was detected in fetal lacrimal gland tissue, but at much lower levels in adult lacrimal glands (Figure 2). We show further that the expression of *Aqp*3 mRNA was relatively high in the adult murine cornea and conjunctiva. AQP3, which functions as a water, glycerol, and hydrogen peroxide transporting channel, was particularly highly expressed in the basal layer of the epidermis of skin keratinocytes and had an important role in skin hydration, nutrition, wound healing, and barrier repair [46]. In corneal epithelial cells, AQP3 had been shown to accelerate corneal wound healing by interacting with phospholipase D2 [47].

The development of the murine lacrimal gland begins on E13.5 as a bud formed on the conjunctival epithelium [22]. Therefore, these data suggest that the loss of AQP3 expression indicates normal development of the lacrimal gland from the conjunctiva. In the lacrimal gland, AQP4 is a major protein expressed in the basolateral membranes of acinar and duct cells [12]. Using qPCR analysis, we found here that lower levels of *Aqp*4 mRNA were expressed in adult lacrimal glands than *Aqp*8 and *Aqp*9 mRNA (Figure 1). The differences between the present study and those of others may be attributed to the detection methods and species employed.

In the present study, we found that AQP8, AQP9, and *α*-SMA were coexpressed in myoepithelial cells surrounding the lacrimal gland acinar units. Elkjaer et al. detected AQP8 in the myoepithelial cells of the salivary, bronchial, and tracheal glands [48]. Myoepithelial cells are typically present in the glandular epithelium as a thin layer above the basement membrane, and evidence indicates that they contract to induce the secretion of products from the acinar cells into the ducts [49]. Moreover, adult lacrimal glands contain progenitor cells of the myoepithelium that express *α*-SMA [50]. In a murine model of impaired secretory function of the lacrimal gland induced by interleukin-1, the lacrimal gland regenerates and cell function is rapidly restored and is accompanied by proliferation of acinar and myoepithelial cells [51], suggesting that myoepithelial cells arise from progenitor cells of lacrimal glands. However, the physiological role of AQP8 remains to be determined.

In conclusion, we show here multiple AQP family members are expressed in the lacrimal gland at discrete developmental stages (Table 1). These findings provide a foundation for future studies devoted to defining the respective roles of each AQP in the homeostasis and secretory function of the lacrimal glands. We speculate that multiple AQPs may provide compensatory and parallel pathways for differential or region-specific regulation of water transport in the lacrimal glands. Among them, AQP8 was the most specific expression in mRNA and protein level. Such cell-type and tissue-specific localization of AQP family members in lacrimal glands suggests that further studies of AQPs will illuminate the precise mechanisms of lacrimal gland development and secretory function.

## 4. Methods

### 4.1. Preparation of Murine Tissues

All procedures undertaken in the present study conformed to the principles outlined in the *Guide for the Care and Use of Laboratory Animals* published by the USA National Institutes of Health (NIH Publication No. 85-23, revised 1996) and were approved by the Institutional Animal Care and Use Committee of Keio University School of Medicine (permission No. 08067-2). All animals were handled in full accordance with the ARVO Statement for the Use of Animals in Ophthalmic and Vision Research and institutional guidelines.

C57BL/6 mice were purchased from CLEA Japan, Inc., Tokyo, Japan. Mice were sacrificed, and lacrimal gland tissues were removed from E13.5 (*n* = 3; unknown gender) and E17.5 (*n* = 3, unknown gender) embryos and adult male mice (8 weeks of age, *n* = 3). Corneal and conjunctival tissues were also obtained from adult mice (*n* = 3). Because embryo samples were too small, each quantitative qPCR was performed with the pool of 20 embryos, and 3 samples (*n* = 3, 60 embryos) were analyzed in this study. For adult male mice, each sample was analyzed (*n* = 3). For qPCR, samples were immediately treated with RNAlater® (QIAGEN, Hilden, Germany) according to the manufacturer's instructions. For histopathological analysis, samples were immediately embedded in the optimal cutting temperature (OCT) compound (Tissue-Tek; Miles Inc., Elkhart, IN) and stored at −80°C.

### 4.2. Quantitative Real-Time PCR (qPCR)

Total cellular RNA from murine tissues was isolated using ISOGEN (Nippon Gene, Hilden, Germany) and purified using the RNeasy® Mini Kit (Qiagen) and DNase I (Qiagen) according to the manufacturer's specifications. One microgram of total RNA was converted to cDNA (iScript cDNA Synthesis kit; BioRad) and used as a template. Quantitative real-time PCR was performed using SYBR® Green I, which binds double-stranded DNA, using a Step One plus (Life Technologies, Carlsbad, CA). The expression levels of mRNAs were normalized to the median level of a housekeeping gene (GAPDH). The copy number is expressed as the number of transcripts/ng total RNA. The primer sequences for murine *Gapdh* and *Aqp*0-*Aqp*9, *Aqp*11, and *Aqp*12 are shown in Table 2.

### 4.3. Immunofluorescence Assay (IFA)

Immunofluorescence assay was performed as described previously. Briefly, frozen sections of lacrimal glands and whole eye tissues (5 *μ*m thick) were air-dried, fixed in cold acetone for 10 minutes, and blocked with 5% donkey serum in phosphate-buffered saline (PBS) for 30 min. Sections were then incubated overnight at 4°C with the primary antibodies as follows: rabbit anti-AQP3 polyclonal antibody (sc-20811, H-80, Santa Cruz Biotechnology, Inc., Santa Cruz, CA); rabbit anti-AQP5 polyclonal antibody (sc-28628, H-200, Santa Cruz); rabbit anti-AQP8 (sc-28624, H-85, Santa Cruz); rabbit anti-AQP9 (ab105148, Abcam, Cambridge, MA); and goat anti-*α*-SMA polyclonal antibody (ab21027, Abcam). The sections were next incubated for 30 min with the species-appropriate secondary antibodies as follows: AlexaFlour® 488-conjugated donkey anti-goat IgG (Life Technologies, Carlsbad, CA) and AlexaFlour® 555-conjugated anti-rabbit goat IgG (Life Technologies) and then washed with PBS and mounted using Permafluor (Beckman Coulter Inc., Miami, FL). Images were acquired using an Axioplan 2 microscope (Carl Zeiss Inc., Thornwood, NY) equipped with a digital camera (Axiocam; Carl Zeiss Inc.).

### 4.4. Statistical Analysis

Differences between groups were analyzed using one-way analysis of variance (ANOVA) or Kruskal-Wallis with Dunn's post hoc testing to analyze the qPCR data. A *p* value of less than 0.05 was considered significant. Values are expressed as the mean ± standard deviation (SD).

## Figures and Tables

**Table 1:** Summary of *Aqp* gene expression in murine lacrimal gland (E13.5d, E17.5d, and adult) and adult cornea and conjunctiva.

	*Aqp*0	*Aqp*1	*Aqp*2	*Aqp*3	*Aqp*4	*Aqp*5	*Aqp*6	*Aqp*7	*Aqp8*	*Aqp*9	*Aqp*11	*Aqp*12
Lacrimal gland (E13.5d)	−	−	−	+/−	−	−	−	−	−	−	−	−
Lacrimal gland (E17.5d)	−	−	−	−	−	−	−	−	−	−	−	−
Lacrimal gland (8 w)	−	−	−	−	+/−	++	−	−	+++	++	+/−	−
Conjunctiva (8 w)	−	−	−	+++	+	+/−	−	−	−	−	−	−
Cornea (8 w)	−	+	−	+++	−	+++	−	−	−	−	+/−	−

−: raw data≦1000 copies; +: raw data > 1000 copies; ++: raw data > 3000 copies; +++: raw data > 10000 copies.

**Table 2:** qPCR primers used in this research.

Gene	Primer sequence (5′-3′)	
	Forward	Reverse
*Aqp0*	ATCCTCACCCGGAACTTCA	GGGGGAAGAGGAGAAAGTCA
*Aqp1*	CCTCCAGGCACAGTCTTCTC	CAGTGGCCTCCTGACTCTTC
*Aqp2*	TCCTTCGAGCTGCCTTCTAC	CGGCTGTTGCATTGTTGT
*Aqp3*	GCCCTCCAGAATTTCTATGAACTCT	TTTGCTATCCTACCTTGGCTTAAAG
*Aqp4*	GGGAGAGGTATTGTCTTCCGTATG	ATGGGTGGCAGGAAATCTGA
*Aqp5*	TGCTCCGAGCCATCTTCTAC	TTGCCTGGTGTTGTGTTGTT
*Aqp6*	TATCTCTCTGCCTCGGGCTA	TCCATGGAAGCAAAGACACA
*Aqp7*	AGGTCTGTGCTGGAGACCAT	CCAAAACCCAAGTTGACACC
*Aqp8*	TGTGTAGTATGGACCTACCTGAG	ACCGATAGACATCCGATGAAGAT
*Aqp9*	AGAAACCCCAAGATGCCTTC	ACCAGCCTTTTCTCGACTGA
*Aqp11*	TAGCTTGCAGGAATCCCATC	CGCTGGGTTAAACAATGCTC
*Aqp12*	GGGAACACCTTGCTGGAGTA	ACTCCCCTTTGTGCATCTTG
*Gapdh*	TGACGTGCCGCCTGGAGAAA	AGTGTAGCCCAAGATGCCCTTCAG

## Data Availability

The gene and immunofluorescence data used to support the findings of this study are included within the article.
